# Achilles Tendon Rupture and Dysmetabolic Diseases: A Multicentric, Epidemiologic Study

**DOI:** 10.3390/jcm11133698

**Published:** 2022-06-27

**Authors:** Francesco Oliva, Emanuela Marsilio, Giovanni Asparago, Alessio Giai Via, Carlo Biz, Johnny Padulo, Marco Spoliti, Calogero Foti, Gabriella Oliva, Stefania Mannarini, Alessandro Alberto Rossi, Pietro Ruggieri, Nicola Maffulli

**Affiliations:** 1 Department of Medicine, Surgery and Dentistry, University of Salerno, Via S. Allende, 84081 Baronissi, Italy; olivafrancesco@hotmail.com (F.O.); giovanni.asparago@gmail.com (G.A.); n.maffulli@qmul.ac.uk (N.M.); 2 Department of Orthopaedic Surgery and Traumatology, San Camillo-Forlanini Hospital, 00152 Rome, Italy; alessiogiaivia@hotmail.it; 3 Orthopaedics and Orthopaedic Oncology, Department of Surgery, Oncology and Gastroenterology DiSCOG, University of Padua, 35128 Padova, Italy; carlo.biz@unipd.it (C.B.); pietro.ruggieri@unipd.it (P.R.); 4 Department of Biomedical Sciences for Health, Università degli Studi di Milano, 20133 Milan, Italy; johnny.padulo@unimi.it; 5 Orthopaedics and Traumatology Unit, Department of Emergency and Acceptance, Azienda Ospedaliera San Camillo-Forlanini, 00152 Rome, Italy; marcodoc@me.com; 6 Physical and Rehabilitation Medicine, Clinical Sciences and Translational Medicine Department, Tor Vergata University, 00133 Rome, Italy; foti@med.uniroma2.it; 7 Department of Internal Medicine, Ospedale del Mare, ASL1, 80147 Napoli, Italy; gably@libero.it; 8 Department of Philosophy, Sociology, Education, and Applied Psychology, Section of Applied Psychology, University of Padova, 35128 Padova, Italy; stefania.mannarini@unipd.it (S.M.); a.rossi@unipd.it (A.A.R.); 9 Interdepartmental Center for Family Research, University of Padova, 35128 Padova, Italy; 10 School of Pharmacy and Bioengineering, Keele University Faculty of Medicine, Thornburrow Drive, Stoke on Trent ST4 7QB, UK; 11 Centre for Sports and Exercise Medicine, Barts and the London School of Medicine and Dentistry, Mile End Hospital, Queen Mary University of London, 275 Bancroft Road, London E1 4DG, UK

**Keywords:** Achilles tendon, rupture, dysmetabolism, diabetes mellitus, thyroid disease, hypercholesterolemia, obesity, surgery, rehabilitation

## Abstract

**Introduction:** Achilles tendon ruptures are common. Metabolic disorders, such as diabetes mellitus, hypercholesterolemia, thyroid disorders, and obesity, impair tendons health, leading to Achilles tendinopathy and likely predisposing patients to Achilles tendon ruptures. **Materials and methods:** Patients who visited the Orthopedic Outpatient Clinics and the Accident and Emergency Departments of five different hospitals in Italy were recruited. Through telephone interviews, we administered a questionnaire to all the patients who had undergone surgical ATR repair, evaluating their past medical history, sport- and work-related activities, drug use, and post-operative rehabilitation outcomes. **Results:** “Return to work activities/sport” was negatively predicted by the presence of a metabolic disorder (β = −0.451; OR = 0.637) and ‘open’ surgery technique (β = −0.389; OR = 0.678). “Medical complications” were significantly predicted by metabolic disorders (β = 0.600 (0.198); OR = 1.822) and was negatively related to ‘mini-invasive’ surgery (i.e., not ‘open’ nor ‘percutaneous’) (β = −0.621; OR = 0.537). “Immediate weightbearing” and “immediate walking without assistance” were negatively predicted by ‘open’ technique (β = −0.691; OR = 0.501 and β = −0.359 (0.174; OR = 0.698)). **Conclusions:** Metabolic conditions can strongly affect post-operative outcomes following surgical repair of acute Achilles tendon tears.

## 1. Introduction

The Achilles tendon is the strongest and largest tendon of the human body, and it is the most frequently ruptured, with an increased frequency in both recreational and competitive sports [1]. In 85% of patients, the rupture occurs 2–7 cm proximal to its calcaneal insertion [2]. When diagnosed and treated within six weeks, Achilles tendon ruptures (ATRs) are classified as acute. These ruptures show an increasing incidence that varies from 11 to 37/100,000 in the European population, and they usually occur in active young and adult patients aged between 37 and 44 years [3]. ATRs usually occur more frequently in men than women, with male–female ratios from 1.7:1 to 30:1 [4,5,6]. ATRs can lead to persistent disability and functional impairment and can compromise patients’ return to sport and everyday activities [7]. The current management of ATRs aims to restore function and avoid complications, either surgically or non-surgically [8,9]. Open, percutaneous, and mini-invasive techniques are all advocated as surgical options, even though conservative treatment has shown no significant differences compared to operative management [8,10,11]. The etiology of ATRs is multifactorial, and several intrinsic and extrinsic risk factors can be identified. While age, sex, and systemic conditions are non-modifiable predictors of rupture, BMI, use of different types of drugs, and sport involvement represent modifiable risk factors [12,13,14]. Metabolic diseases, such as diabetes mellitus, hypercholesterolemia, thyroid disorders, and obesity, impair tendon health, leading to Achilles tendinopathy and predisposing one to ATR [15]. Recently, the effects of metabolic disorders on tendons have been studied; these conditions modify the histology, mechanical structure, and healing capabilities of the tendons [16,17,18]. However, the association between these conditions and ATR has not yet been clarified.

The purposes of the present study were first to observe the incidence of metabolic diseases in patients with an ATR, and then to identify any significant differences in terms of patients’ medical records, surgical approaches used, rehabilitation, and return to sport and/or everyday life activities.

## 2. Material and Methods

We retrospectively surveyed patients who had attended the Orthopedic Outpatient Clinics and the Accident and Emergency Departments of five different hospitals in Italy for ATRs: Ospedali Riuniti San Giovanni di Dio e Ruggi d’Aragona, Salerno, Tor Vergata University Hospital, Rome, University Hospital of Padova, San Camillo-Forlanini Hospital, Rome, and Sant’Anna Hospital, Como. Complete medical histories were collected. Then, through telephone interviews, we administered a questionnaire to all of the patients who had been surgically treated for ATRs (Table 1). The inclusion criteria were that (1) patients had suffered an ATR and (2) had undergone surgery between January 2003 and December 2021. Patients who suffered from a head trauma or coma and patients who did not consent to data treatment were excluded. Personal data of the patients were recorded (sex, age, and BMI). Then, comorbidities of the patients were investigated, especially the patients’ medical history of type I (T1DM) or II diabetes mellitus (T2DM), obesity, thyroid disease (hypothyroidism and hyperthyroidism), and hypercholesterolemia. The diagnosis of Achilles tendon rupture was performed by a fully trained orthopedic surgeon using clinical tests (palpation, calf squeeze test, knee flexion test). The present study was approved and registered by the ethics committee of the University of Salerno (project ID 0096390, 19 June 2020) and was performed according to the principles expressed in the Declaration of Helsinki. Descriptive statistics are reported in Table 2 and Table 3.

## 3. Statistical Analysis

Statistical analyses were performed with the R software (R-core project [19,20]) and the following packages: psych [21], lme4 [22], lavaan [23,24], semPlot, and ggplot2.

A multiple-multivariate binary logistic regression (‘*probit*’ link function) was used to investigate the association between literature-based predictors and four literature-relevant outcomes evaluated in patients with Achilles tendon tears. In more detail, the dependent binary variables (outcomes) were (A) ‘Return to sport/work related activities’ (no/yes), (B) ‘Medical complications’ (no/yes), (C) ‘Immediate weightbearing’ (no/yes), and (D) ‘Immediate walking without assistance’ (no/yes). On the contrary, independent variables (predictors) were: (A) ‘Familiar history’ (no/yes), (B) ‘Past agonistic sport’ (no/yes), (C) ‘Dysmetabolism’ (no/yes), (D) ‘BMI’, € ‘Surgery: ‘open’’ (no/yes), (F) ‘Surgery: ‘percutaneous’’ (no/yes), and (G) ‘Age of surgery’ (≤50 y.o./>50 y.o.). The *p*-value was set to 0.05. All reported beta coefficients and odds ratios are unstandardized.

## 4. Results

The medical records of 500 patients surgically treated for Achilles tendon rupture were collected from five different hospitals in Italy from 2003 to 2021. Of those, 160 patients did not consent to the post-operative interviews, and therefore were not part of the present study. The remaining 340 patients were allocated to one of two groups based on a medical history of metabolic diseases: 95 patients (28%) had a medical record of diabetes mellitus, obesity, hypercholesterolemia, and/or thyroid conditions, and they were allocated to the dysmetabolic group; 245 patients (72%) who had no history of metabolic diseases were allocated to the non-dysmetabolic group. The baseline demographic characteristics of the population are reported in Table 2. Further details about the frequency and baseline characteristics of the dysmetabolic patients are shown in Table 3.

The first outcome of the multiple-multivariate logistic regression, “*Return to work activities/sport*”, was negatively predicted by dysmetabolism (β = −0.451 (0.164), 95% CI_β_: from −0.771 to −0.130; OR = 0.637, 95% CI_OR_: from 0.462 to 0.878) and ‘open’ surgery technique (β = −0.389 (0.179), 95% CI_β_: from −0.740 to −0.038; OR = 0.678, 95% CI_OR_: from 0.477 to 0.962), while it was positively predicted by age at which the surgery was performed (β = 0.629 (0.167), 95% CI_β_: from 0.300 to 0.957; OR = 1.875, 95% CI_OR_: from 1.350 to 2.603). The explained variance (*R*^2^) was equal to 0.131. The second outcome of the multiple-multivariate logistic regression, “*Medical complications*”, was significantly predicted by dysmetabolism (β = 0.600 (0.198), 95% CI_β_: from 0.211 to 0.989; OR = 1.822, 95% CI_OR_: from 1.235 to 2.688), while it was negatively related to ‘mini-invasive’ surgery (i.e., not ‘open’ nor ‘percutaneous’) (β = −0.621 (0.253), 95% CI_β_: from −1.117 to −0.125; OR = 0.537, 95% CI_OR_: from 0.327 to 0.883). The explained variance (*R*^2^) was 0.097. The third outcome of the multiple-multivariate logistic regression, “*Immediate weightbearing*”, was significantly predicted by past competitive sport experiences (β = 0.396 (0.169), 95% CI_β_: from 0.063 to 0.728; OR = 1.485, 95% CI_OR_: from 1.066 to 2.070) and age at which the surgery was performed (β = 0.342 (0.171), 95% CI_β_: from 0.007 to 0.676; OR = 1.407, 95% CI_OR_: from 1.007 to 1.967), while it was negatively predicted by ‘open’ technique (β = −0.691 (0.205), 95% CI_β_: from −1.093 to −0.288; OR = 0.501, 95% CI_OR_: from 0.335 to 0.749). The explained variance (*R*^2^) was equal to 0.125. Lastly, the first outcome of the multiple-multivariate logistic regression, “*Immediate walking without assistance*”, was significantly predicted by past competitive sport experiences (β = 0.406 (0.148), 95% CI_β_: from 0.115 to 0.697; OR = 1.501, 95% CI_OR_: from 1.122 to 2.007) but negatively predicted by ‘open’ surgery technique (β = −0.359 (0.174), 95% CI_β_: from −0.700 to −0.019; OR = 0.698, 95% CI_OR_: from 0.497 to 0.981). The explained variance (*R*^2^) was 0.065. All results are reported in detail in Table 4 and Table 5.

## 5. Discussion

This study showed that some metabolic conditions can strongly affect the post-operative outcome of patients who have undergone surgery to repair an acute Achilles tendon tear. Ninety-five patients (28%) had a medical history of metabolic pathologies. Of them, hypercholesterolemia (40 patients, 42%) and obesity (22 patients, 23%) were the most frequently reported, followed by thyroid disorders, such as hypothyroidism, hyperthyroidism, and Hashimoto disease (17 patients, 18%), as well as diabetes mellitus (16 patients, 17%). The multivariate statistical analysis showed that the diagnosis of a dysmetabolic disease significantly predicted the risk of post-operative complications and a delayed return to sport and work activities (Table 4 and Table 5).

Hypercholesterolemia was the most common metabolic condition in our study population of patients with ATRs. Serum cholesterol is carried in various form, of which LDL cholesterol (LDL-C) is the most atherogenic [25]. Indeed, patients with high levels of total cholesterol (>200 mg/dL; 5.17 mmol) and LDL cholesterol (>100 mg/dL; 2.58 mmol) can be diagnosed with hypercholesterolemia [26]. In addition, 1/500 people per year can present with familial hypercholesterolemia (HeFH), which arises from the heterozygous mutation of the gene that codifies for the LDL receptor [27]. The lack of this receptor, which usually removes LDL from the serum, leads to higher levels of blood LDL cholesterol [28]. High levels of serum lipids can negatively affect tendon homeostasis [29,30]. The Achilles tendon structure is characterized by the presence of type I collagen, which accounts for 95% of the tendon dry weight [31]. High levels of LDL-C increase the metalloproteinase (MMP) activity, eliciting a chronic inflammatory response and impacting the tendon structure [30]. In a recent retrospective study, lipid blood levels were compared among patients who suffered an ATR and healthy controls. Yang et al. [18] found that serum cholesterol, triglyceride, and LDL levels in patients who ruptured their Achilles tendon were significantly higher than those in healthy controls [18]. The low-grade chronic inflammation found in patients with high serum cholesterol levels is commonly reported in obese subjects as well. Obesity is a modifiable risk factor for several musculoskeletal conditions [32]. The World Health Organization recognized BMI values over 30 kg/m^2^ as a cutoff value for adult obesity, while other measurements, such as the waist and hip ratio, have been used to define the pattern of fat distribution [33]. Adipose tissue releases hormones and proteins that influence tenocyte function and homeostasis [34]. Adipokines play a crucial part in this molecular pathway, causing an increase in MMP activity and the migration of macrophages and mast cells to the adipose tissue, leading to a reduction in their homeostatic functions [35,36]. As a result, a chronic inflammatory state, and alterations in the type I/type III collagen synthesis result in the tendon being less resistant to stress and more at risk of rupture [37]. The role of mechanical loads on weightbearing tendons in obese patients is still debated. Indeed, obesity is associated with a high risk of tendon tears, even in the upper extremities, such as rotator cuff tears [38,39]. A recent systematic review compared the relationship of being overweight and suffering from tendon tears: Sedentary lifestyle was identified as a risk factor for rupture, while mechanical loads are responsible for pain and tendon inflammation [40]. Increased abdominal adiposity, chronic inflammation status, and endocrine system disfunction are also three of the main features of diabetes mellitus [41]. Type 2 diabetes mellitus (T2DM) is a public health challenge because of its clinical and economical burdens on the worldwide population [42]. In the US alone, nearly 10% of the population has been diagnosed with T2DM, while 83 million people have been classified as pre-diabetic with a high risk of disease progression in 5 years [43,44]. The effects of diabetes mellitus on tendons have been investigated in animal and human models, finding that the crosslink reaction between collagens and advanced glycosylation end-products (AGEs)—highly induced by a persistent state of hyperglycemia—is accountable for the disruption of tendon homeostasis [45,46,47]. Zakaria et al. investigated the incidence and predictors of tendon rupture requiring hospitalization in patients affected by T2DM versus healthy controls, identifying a significantly higher risk of tendon rupture in diabetic subjects [48]. In addition, the structural setting of tendons can be significantly altered by thyroid hormones [49], as shown by in vitro and in vivo studies in animal and human tendons [50,51,52,53,54,55,56]. Oliva et al. [57] investigated the healing capabilities of the combination of T3 and ascorbic acid (AA) compared to the administration of bone marrow mesenchymal stem cells alone in a rat model of Achilles tendon injury. The association of T3 and AA can improve Achilles tendon healing in terms of the type I/type III collagen ratio, tendon stiffness, and strength [57]. The current evidence shows that metabolic diseases, such as T2DM and thyroid diseases, are not only risk factors for tendon ruptures, but also predictive of delayed post-operative healing [52,58,59]. In the current study, we investigated how long it took for dysmetabolic and healthy patients to return to sport and work activities; the presence of any metabolic condition led to a delayed return to both sport and work activities. Dombrowski et al. [60] investigated the role of comorbidities in the development of post-operative complications, showing a significant relationship between obesity or T2DM and surgical site infections [61]. In accordance, our study showed that metabolic conditions can be regarded as positive predictive factors for post-operative complications, such as deep and superficial surgical site infection and delayed wound healing. Even though there is no consensus about the gold-standard strategy for treating acute ATRs, it appears that the choice of the surgical technique can play a role in the assessment of risk for post-operative complications. Recently, a randomized superiority trial in 39 UK hospitals compared surgical and conservative treatments after an ATR. The main outcomes of interest were the Achilles tendon rupture score (ATRS) and the re-rupture rate in both groups; patients treated non-surgically experienced the same results as those in the surgically treated group [10]. Consistently with the results reported by Del Buono et al. [62], we found that choosing a ‘mini-invasive’ over an ‘open’ surgical approach is associated with a significant reduction in post-operative complications. The surgical technique used can influence the rehabilitation protocol in terms of immediate weightbearing and walking without assistance. Indeed, open techniques were associated (*p* < 0.001) with a statistically significant longer time of weightbearing (>40 days) and walking (>3 months) in both dysmetabolic and healthy patients. We also investigated the relationship between patients’ participation in competitive sports and the surgical treatment of ATRs, finding no statistically significant differences between athletes and non-athletes. Even though the relationship between ATRs and systemic conditions has been investigated [63,64], the current investigation is the first study in the Italian population to investigate the influence of T2DM, thyroid diseases, hypercholesterolemia, and obesity on acute Achilles tendon ruptures. Obviously, this study has some limitations. First, the small number of patients with metabolic conditions could be related to the relatively young age range at which an ATR occurs. Indeed, patients with a family history of metabolic diseases could develop the condition later than the tendon rupture. The surgeons who took part in the study did not use the same surgical techniques and did not follow the same rehabilitation protocols, which could lead to a higher risk of bias. However, this reflects the standard practice in each of the centers involved in the present study and allows greater generalization of the results. Retrospective assessment of demographic data was carried out through telephone interviews, leading to an inevitable lack of relevant medical information. Furthermore, the questionnaire used to investigate the medical histories of the patients has not yet been validated. This limitation could increase the risk of bias in the study, and it could represent the aim of further studies to achieve more scientifically valid findings.

## 6. Conclusions

Metabolic conditions can strongly affect the post-operative outcomes of surgical reconstruction of the Achilles tendon.

## Figures and Tables

**Table 1:** Telephone questionnaire administered to the participants. Note: BMI = Body Mass Index; HRT = Hormone Replacement Therapy.

Name and Surname:		
TELEPHONE QUESTIONNAIRE		

Patients admitted to __________________________, who underwent an Achilles tendon reconstruction surgery during the period__________________________. The data collection took place in the period _____________________. The questionnaire consists of **26 questions**, of which **11** have dichotomous answers (YES/NO), **10** have multiple answers, and **5** have open answers.		

Anthropometric data of the patient:		
	- weight: kg	
	- height: cm	
	- BMI:	

QUESTIONS:		
1. If a woman, **Menopause**?		YES __NO __
2. If yes, take HRT?		YES __NO __
3. What is your work activity?		

**4. Type of rupture of the Achilles tendon?**		
	- unilateral	__
	- bilateral	__
	- total	__
	- re-rupture	__

**5. Do you have a family history of tendon rupture/tendinopathies?**		
	- father	__
	- mother	__
	- sister	__
	- brother	__
	- uncle	__
	- aunt	__
	- cousin	__
	- female cousin	__
	- NO/is not aware	__

6. Are you taking any drugs?		YES __NO __
7. If so, which drugs?		

**8. Before the Achilles tendon rupture, have you had targeted therapy with antibiotics (quinolones), steroids, or others?**		YES __NO __
9. If so, which drugs?		

**10. Do you suffer from any noteworthy pathologies?**		YES __NO __
11. If so, which pathologies?		
	- hypercholesterolemia	__
	- diabetes mellitus	__
	- hyperthyroidism	__
	- hypothyroidism	__
	- obesity	__
**12. Have you ever suffered from tendon conditions?**		YES __NO __
13. If so, which tendons?		

**14. Have you ever suffered from localized problems at the Achilles tendon level?**		YES __NO __
15. Which type of these:		
	- tendon rupture (relapse)	__
	- Achilles peritendinitis	__
	- Achilles tendinosis	__
	- Achilles insertional tendinopathy	__
	- calcaneal apophysite (Sever’s disease)	__
	- other	__

**16. Do you practice sports?**		YES __ NO __
17. If so, what kind?		
	- competitive	__
	- not competitive	__

**18. What kind of strain/overload does it exert on the muscle–tendon apparatus during work?**		
	- none	__
	- light	__
	- moderate	__
	- intense	__

**19. Did you engage in strenuous exercise or overload stress prior to the tendon rupture?**		YES __NO __

**20. The surgery was performed with the following technique:**		
	- open	__
	- minimally invasive	__
	- percutaneous	__

**21. Were there any complications after surgery?**		YES __NO __
22. If so, what kind?		
	- superficial infections	__
	- deep tissue infections	__
	- surgical wound repair defects	__

**23. How long did it take to start loading?**		
	- immediately	__
	- 6 weeks after surgery	__
	- after 6 weeks	__

**24. How long after were you able to walk independently without any help?**		
	- 1 month	__
	- 2 months	__
	- 3 months	__

**25. How long did it take for you to start sports?**		
	- after 6 months	__
	- 12 months	__

**26. Do you think you have returned to the previous functional sport/work level?**		YES __NO __

**Table 2:** Baseline demographic characteristics. Note: ATR = Achilles Tendon Rupture; BMI = Body Mass Index; SD = Standard Deviation.

	Patients with ATR (n)	Female (%)	Male (%)	BMI (Mean)	Mean Age (SD)
**Total**	340	59	281	26.15	45 (±13.06)
**Dysmetabolic**	95	27	68	26.87	51 (±14.19)
**Non-Dysmetabolic**	245	32	213	25.87	43 (±11.77)

**Table 3:** Baseline characteristics of the dysmetabolic group.

	Diabetes Mellitus	Thyroid Diseases	Obesity	Hypercholesterolemia
**Patients (n)**	16	17	22	40
**Male (%)**	9 (56%)	2 (12%)	18 (82%)	39 (98%)
**Female (%)**	7 (44%)	15 (88%)	4 (8%)	1 (2%)
**Mean Age (years)**	48	60	50	51

**Table 4:** Multiple multivariate logistic regression. Note: OUTC. = outcome variable; PRED. = predictor variable; β* = standardized beta coefficient; β = unstandardized beta coefficient; s.e. = standard error; 95% CI [L-U] = lower and upper 95% confidence interval; BMI = Body Mass Index.

	Variable		β*	β (s.e.)	95% CI [L-U]	*z*-Value	*p*-Value	OR	95% CI [L-U]
**OUTC.**	**Return to work/sport activities**								
**PRED.**	**Familiarity**	a1	−0.021	−0.068 (0.209)	[−0.477; 0.341]	−0.327	0.744	0.934	[0.621; 1.406]
**PRED.**	**Past agonistic sport**	b1	0.123	0.268 (0.151)	[−0.027; 0.563]	1.779	0.075	1.307	[0.973; 1.756]
**PRED.**	**Dysmetabolism**	c1	−0.189	−0.451 (0.164)	[−0.771; −0.130]	−2.758	0.006	0.637	[0.462; 0.878]
**PRED.**	**BMI**	d1	0.046	0.016 (0.024)	[−0.032; 0.064]	0.648	0.517	1.016	[0.968; 1.066]
**PRED.**	**Surgery ‘open’**	e1	−0.178	−0.389 (0.179)	[−0.740; −0.038]	−2.174	0.030	0.678	[0.477; 0.962]
**PRED.**	**Surgery ‘percutaneous’**	f1	−0.069	−0.156 (0.188)	[−0.525; 0.213]	−0.830	0.407	0.855	[0.591; 1.237]
**PRED.**	**Surgery ‘mini-invasive’**	g1	0.053	0.131 (0.207)	[−0.275; 0.537]	0.630	0.529	1.139	[0.759; 1.710]
**PRED.**	**Age of surgery**	h1	0.274	0.629 (0.167)	[0.300; 0.957]	3.754	<0.001	1.875	[1.350; 2.603]
									
**OUTC.**	**Medical complications**								
**PRED.**	**Familiar history**	a2	0.109	0.355 (0.256)	[−0.146; 0.857]	1.389	0.165	1.426	[0.864; 2.355]
**PRED.**	**Past agonistic sport**	b2	0.080	0.171 (0.188)	[−0.197; 0.539]	0.911	0.362	1.187	[0.821; 1.714]
**PRED.**	**Dysmetabolism**	c2	0.256	0.600 (0.198)	[0.211; 0.989]	3.023	0.002	1.822	[1.235; 2.688]
**PRED.**	**BMI**	d2	−0.008	−0.003 (0.027)	[−0.056; 0.050]	−0.101	0.919	0.997	[0.946; 1.052]
**PRED.**	**Surgery ‘open’**	e2	−0.122	−0.261 (0.205)	[−0.663; 0.141]	−1.271	0.204	0.770	[0.515; 1.152]
**PRED.**	**Surgery ‘percutaneous’**	f2	−0.142	−0.313 (0.241)	[−0.785; 0.159]	−1.300	0.193	0.731	[0.456; 1.172]
**PRED.**	**Surgery ‘mini-invasive’**	g2	−0.255	−0.621 (0.253)	[−1.117; −0.125]	−2.452	0.014	0.537	[0.327; 0.883]
**PRED.**	**Age of surgery**	h2	−0.046	−0.105 (0.197)	[−0.491; 0.282]	−0.530	0.596	0.901	[0.612; 1.326]
									
**OUTC.**	**Immediate weightbearing**								
**PRED.**	**Familiar history**	a3	0.107	0.356 (0.229)	[−0.093; 0.806]	1.554	0.120	1.428	[0.911; 2.238]
**PRED.**	**Past agonistic sport**	b3	0.182	0.396 (0.169)	[0.063; 0.728]	2.335	0.020	1.485	[1.066; 2.070]
**PRED.**	**Dysmetabolism**	c3	−0.085	−0.203 (0.184)	[−0.565; 0.158]	−1.103	0.270	0.816	[0.568; 1.171]
**PRED.**	**BMI**	d3	0.054	0.019 (0.025)	[−0.029; 0.067]	0.760	0.447	1.019	[0.971; 1.069]
**PRED.**	**Surgery ‘open’**	e3	−0.318	−0.691 (0.205)	[−1.093; −0.288]	−3.365	0.001	0.501	[0.335; 0.749]
**PRED.**	**Surgery ‘percutaneous’**	f3	−0.053	−0.119 (0.202)	[−0.516; 0.277]	−0.590	0.555	0.887	[0.597; 1.319]
**PRED.**	**Surgery ‘mini-invasive’**	g3	−0.102	−0.252 (0.193)	[−0.631; 0.126]	−1.307	0.191	0.777	[0.532; 1.134]
**PRED.**	**Age of surgery**	h3	0.149	0.342 (0.171)	[0.007; 0.676]	1.999	0.046	1.407	[1.007; 1.967]
									
**OUTC.**	**Immediate walking without assistance**								
**PRED.**	**Familiarity**	a4	−0.035	−0.111 (0.219)	[−0.541; 0.318]	−0.508	0.611	0.894	[0.582; 1.375]
**PRED.**	**Past agonistic sport**	b4	0.193	0.406 (0.148)	[0.115; 0.697]	2.734	0.006	1.501	[1.122; 2.007]
**PRED.**	**Dysmetabolism**	c4	−0.080	−0.185 (0.161)	[−0.500; 0.130]	−1.150	0.250	0.831	[0.606; 1.139]
**PRED.**	**BMI**	d4	0.033	0.011 (0.023)	[−0.034; 0.056]	0.474	0.636	1.011	[0.966; 1.058]
**PRED.**	**Surgery ‘open’**	e4	−0.171	−0.359 (0.174)	[−0.700; −0.019]	−2.070	0.038	0.698	[0.497; 0.981]
**PRED.**	**Surgery ‘percutaneous’**	f4	−0.130	−0.282 (0.182)	[−0.638; 0.075]	−1.548	0.122	0.755	[0.528; 1.078]
**PRED.**	**Surgery ‘mini-invasive’**	g4	−0.002	−0.004 (0.201)	[−0.399; 0.391]	−0.019	0.985	0.996	[0.671; 1.478]
**PRED.**	**Age of surgery**	h4	0.087	0.192 (0.162)	[−0.125; 0.509]	1.187	0.235	1.211	[0.883; 1.663]

**Table 5:** Covariances (standardized) between dichotomous outcomes of the multiple-multivariate regression analysis. Note: ** *p* = 0.001; *** *p* < 0.001.

	Return to Work/Sport Activities	Medical Complications	Immediate Weightbearing	Immediate Walking without Assistance
**Return to work/sport activities**	-			
**Medical complications**	−0.076	-		
**Immediate weightbearing**	0.065	−0.101	-	
**Immediate walking without assistance**	0.279 **	−0.346 ***	0.561 ***	-
